# “一锅法”制备聚(苯乙烯-丙烯酸)共聚物改性的硅基固定相及其在混合模式液相色谱中的应用

**DOI:** 10.3724/SP.J.1123.2023.01005

**Published:** 2023-07-08

**Authors:** Xiaoqing WANG, Jian CUI, Yiming GU, Shuo WANG, Jin ZHOU, Shudong WANG

**Affiliations:** 1.中国科学技术大学,安徽合肥230026; 1. University of Science and Technology of China, Hefei 230026, China; 2.中国科学院大连化学物理研究所,大连清洁能源国家实验室,辽宁大连116023; 2. Dalian National Laboratory for Clean Energy, Dalian Institute of Chemical Physics, Chinese Academy of Sciences, Dalian 116023, China

**Keywords:** 聚合物改性固定相, 一锅法, 混合模式液相色谱, polymer modified stationary phases, one-pot synthesis, mixed-mode liquid chromatography

## Abstract

聚合物作为一种来源广泛、功能基团丰富、生物相容性良好的修饰配体,被广泛用作硅基色谱固定相。本工作以苯乙烯和丙烯酸为聚合单体,乙烯基三甲氧基硅烷为硅烷偶联剂,通过自由基聚合反应一锅法制备了聚(苯乙烯-丙烯酸)共聚物改性的硅基固定相(SiO_2_@P(St-b-AA))。傅里叶红外光谱、热重分析、扫描电镜、N_2_吸附-脱附、Zeta电势分析等表征手段证明了该固定相已成功合成,且保持了良好的介孔球形结构。在固定相性能评价中,分别采用疏水性分析物、极性分析物和离子型化合物作为探针,对固定相的分离性能和保留机理进行了考察。其中,由于苯环提供的疏水和*π-π*相互作用,烷基苯和多环芳香烃在固定相上的保留随着流动相中甲醇含量的增加而减弱;由于羧酸基团可以为固定相与核苷碱基等极性溶质之间提供亲水相互作用,随着流动相中乙腈含量的增加,极性分析物的保留逐渐增强;与自制的C18和Amide固定相相比,SiO_2_@P(St-b-AA)固定相在反相和亲水模式下均表现出良好的分离性能。此外,通过探究流动相pH对离子型化合物保留的影响,证明了该固定相还具有弱阳离子交换能力。多种分离模式表明SiO_2_@P(St-b-AA)固定相可提供多种相互作用,并在不同极性组分分析物的分离中具有良好的应用前景。通过考察制备方法的重复性,证明了该方法具有良好的制备批次稳定性,简便的“一锅法”为新型聚合物改性硅基固定相的发展提供了一种新思路。

## Abstract (en)

As modified ligands with a wide range of sources, abundant functional groups, and good biocompatibility, polymers have been widely used in the development of silica-based chromatographic stationary phases. In this study, a poly(styrene-acrylic acid) copolymer-modified silica stationary phase (SiO_2_@P(St-b-AA)) was prepared via one-pot free-radical polymerization. In this stationary phase, styrene and acrylic acid were used as functional repeating units for polymerization and vinyltrimethoxylsilane (VTMS) was used as a silane coupling agent to link the copolymer and silica. Various characterization methods, such as Fourier transform infrared (FT-IR) spectroscopy, thermogravimetric analysis (TGA), scanning electron microscopy (SEM), N_2_ adsorption-desorption analysis, and Zeta potential analysis, confirmed the successful preparation of the SiO_2_@P(St-b-AA) stationary phase, which had a well-maintained uniform spherical and mesoporous structure. The retention mechanisms and separation performance of the SiO_2_@P(St-b-AA) stationary phase in multiple separation modes were then evaluated. Hydrophobic and hydrophilic analytes as well as ionic compounds were selected as probes for different separation modes, and changes in the retention of the analytes under various chromatographic conditions, including different methanol or acetonitrile contents and buffer pH values, were investigated. In reversed-phase liquid chromatography (RPLC) mode, the retention factors of alkyl benzenes and polycyclic aromatic hydrocarbons (PAHs) on the stationary phase decreased with increasing methanol content in the mobile phase. This finding could be attributed to the hydrophobic and *π-π* interactions between the benzene ring and analytes. The retention changes of alkyl benzenes and PAHs revealed that the SiO_2_@P(St-b-AA) stationary phase, similar to the C18 stationary phase, exhibited a typical reversed-phase retention behavior. In hydrophilic interaction liquid chromatography (HILIC) mode, as the acetonitrile content increased, the retention factors of hydrophilic analytes gradually increased, and a typical hydrophilic interaction retention mechanism was inferred. In addition to hydrophilic interaction, the stationary phase also demonstrated hydrogen-bonding and electrostatic interactions with the analytes. Compared with the C18 and Amide stationary phases prepared by our groups, the SiO_2_@P(St-b-AA) stationary phase exhibited excellent separation performance for the model analytes in the RPLC and HILIC modes. Owing to the presence of charged carboxylic acid groups in the SiO_2_@P(St-b-AA) stationary phase, exploring its retention mechanism in ionic exchange chromatography (IEC) mode is of great importance. The effect of the mobile phase pH on the retention time of organic bases and acids was further studied to explore the electrostatic interaction between the stationary phase and charged analytes. The results revealed that the stationary phase has weak cation exchange ability toward organic bases and electrostatically repels organic acids. Moreover, the retention of organic bases and acids on the stationary phase was influenced by the analyte structure and mobile phase. Thus, the SiO_2_@P(St-b-AA) stationary phase could provide multiple interactions, as demonstrated by the separation modes described above. The SiO_2_@P(St-b-AA) stationary phase showed excellent performance and reproducibility in the separation of mixed samples with different polar components, indicating that it has promising application potential in mixed-mode liquid chromatography. Further investigation of the proposed method confirmed its repeatability and stability. In summary, this study not only described a novel stationary phase that could be used in RPLC, HILIC, and IEC modes but also presented a facile “one-pot” preparation approach that could provide a new route for the development of novel polymer-modified silica stationary phases.

高效液相色谱(HPLC)已被广泛应用于医药、化学、生物环境等领域^[1⇓-3]^。自1941年Martin和Synge使用硅胶作为色谱填料成功分离了乙酰化氨基酸混合物以来,多种适用于正相液相色谱(NPLC)^[4⇓-6]^、反相液相色谱(RPLC)^[7⇓-9]^、亲水相互作用液相色谱(HILIC)^[10⇓-12]^、离子交换液相色谱(IEC)^[13,14]^等色谱模式的硅基固定相被研发和制备出来。近些年来,随着实际分离样品复杂性的提高,单一的色谱分离模式已经不能满足多种混合样品的分离需求;因此,耦合多种不同性质的配体,制备满足混合模式液相色谱的固定相表现出更大的应用潜力^[15⇓⇓-18]^。

传统色谱固定相的制备通常是在硅胶表面键合各种硅烷偶联剂来实现,这种方法通常存在可使用的硅烷偶联剂种类有限、基团键合密度小、合成过程繁琐等问题^[10,19⇓-21]^。与硅烷偶联剂键合的固定相相比,聚合物改性的固定相由于其键合密度高、单体丰富、制备方法简单等优点被广泛用于新型色谱固定相的开发中,将具有多功能的聚合物或多种聚合单体结合键合到硅胶表面,也成为新型混合色谱固定相的一大研究热点^[22⇓⇓-25]^。例如,娄旭华等^[26]^通过亲核取代反应将聚乙烯马来酸酐键合到氨基硅胶表面,随后进行水解得到了一种弱阳离子交换/亲水相互作用的色谱固定相;Fan等^[27]^以十一碳烯酸和油酸为单体结合4-乙烯基吡啶进行原位聚合,获得了两种亲水/疏水的色谱固定相;Ni等^[28]^则采用表面引发自由基聚合反应成功制备了包括疏水、氢键、离子交换和*π-π*等多种相互作用的咪唑离子液体共聚物固定相。虽然许多研究已经建立起多种聚合物改性硅基固定相的制备方法,但是其制备工艺繁琐,分离、提纯、精制等过程不可避免地会产生环境污染与能源消耗。因此,优化合成工艺、使用更简便的方法开发出具有优良分离性能的新型色谱固定相具有十分重要的意义。

聚丙烯酸常用于对硅胶进行改性以制备亲水色谱固定相,其中羧酸基团除了提供亲水作用外,在不同的pH条件下,还可以为固定相提供阳离子交换作用^[29,30]^。苯乙烯作为疏水性聚合物单体,也常用于制备反相色谱固定相,且制备的固定相具有优异的分离性能^[23]^。在硅胶表面将聚丙烯酸和聚苯乙烯结合起来可能是一种制备混合模式液相色谱固定相的有效方法,但目前还没有这方面的研究报道。受此启发,本工作选取苯乙烯和丙烯酸作为聚合单体,乙烯基三甲氧基硅烷偶联剂作为聚合物在硅胶表面的连接单体,采用简单的自由基聚合方法,“一锅法”合成了聚(苯乙烯-丙烯酸)共聚物改性的硅基固定相(SiO_2_@P(St-b-AA))。分别在反相、亲水和离子交换模式下探讨了固定相的保留机理和分离性能,并评估了所得固定相对混合样品的分离能力和重现性。此外,还对制备方法的批次稳定性进行了考察。

## 1 实验部分

### 1.1 仪器、材料与试剂

Nicolet 6700傅里叶红外光谱仪(Nicolet公司,美国); STA449F3热重分析仪(Netzsch公司,德国); NOVA 2200e介孔物理吸附仪(Quantachrome公司,美国); JSM-7800F场发射扫描电子显微镜(JEOL公司,日本); Zeta电位分析仪(Malvern公司,英国); Agilent 1260型高效液相色谱仪(Agilent公司,美国); Haskel高压装柱机(Haskel公司,美国)。

全多孔硅胶(粒径5 μm,比表面积260 m^2^/g,实验室自制); C18固定相、Amide固定相(粒径5 μm,实验室自制)。乙烯基三甲氧基硅烷偶联剂(VTMS,纯度98%)购于上海阿拉丁生化科技股份有限公司;苯乙烯(St,纯度>99.5%)购于国药集团化学试剂有限公司;丙烯酸(AA,纯度>99%)、偶氮二异丁腈(AIBN,纯度98%)、甲酸铵、甲酸、三乙胺、二甲苯、甲醇均购于上海麦克林生化科技股份有限公司;甲苯、乙苯、苯、萘、苊、芴、尿嘧啶、胞苷、苯甲酸购于国药集团试剂有限公司;丙苯、正丁基苯、戊苯、尿苷、胞嘧啶、乳清酸、对氨基苯甲酸、4-羟基苯甲酸、乙酰水杨酸购于上海麦克林生化科技股份有限公司;茶碱、二羟丙茶碱购于上海贤鼎生物科技有限公司;5-氟尿嘧啶、5-氟胞嘧啶、可可碱、利巴韦林购于上海吉至生化科技有限公司;色谱级甲醇、色谱级乙腈购于美国Spectrum公司;其他试剂均为分析纯。

### 1.2 固定相的制备

首先将5.0 g真空干燥过的硅胶置于100 mL四口烧瓶中,加入30 mL二甲苯搅拌均匀;随后称取3.5 g乙烯基三甲氧基硅烷偶联剂、10.2 g苯乙烯和7.4 g丙烯酸分散于30 mL甲醇中并逐滴加入烧瓶内,滴加完毕后对体系进行加热,升温至65 ℃时,向烧瓶中加入200 mg AIBN,在氮气保护下回流反应24 h;反应结束后,冷却至室温,依次使用二甲苯、甲醇进行洗涤,最后置于60 ℃真空烘箱中干燥过夜,得到聚(苯乙烯-丙烯酸)共聚物改性的硅基固定相(SiO_2_@P(St-b-AA))。合成路线如图1所示。

**图1:**
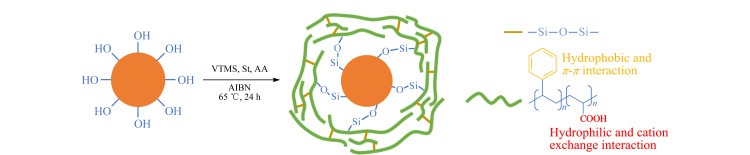
“一锅法”制备SiO_2_@P(St-b-AA)

### 1.3 装柱

称取2.3 g SiO_2_@P(St-b-AA)固定相置于小烧杯中,加入58 mL二氯甲烷-异丙醇(5∶1, v/v)匀浆液,搅拌均匀,超声5 min后,以甲醇为顶替液,在50 MPa压力下将固定相填充于不锈钢色谱柱管中(150 mm×4.6 mm),得到PSA色谱柱。实验室自制的Amide和C18固定相采用相同的方法装填到色谱柱管中,用于与本工作制备的PSA色谱柱进行对比。

### 1.4 色谱条件

流动相为甲醇-水或乙腈-甲酸铵(NH_4_FA)缓冲液。除分离碱性样品时流速为0.5 mL/min外,其他流速均为0.8 mL/min;进样量为1 μL;柱温为30 ℃;烷基苯和多环芳香烃的紫外检测波长为260 nm,其他分析物的紫外检测波长为254 nm。保留因子*k=*(*t-t*_0_)*/t*_0_,其中*t*为分析物的保留时间,*t*_0_为色谱柱的死时间;反相模式下以尿嘧啶在甲醇-水(80∶20, v/v)体系下测定的保留时间作为死时间,亲水模式下以甲苯在乙腈-100 mmol/L甲酸铵(95∶5, v/v)体系下测定的保留时间作为死时间。

## 2 结果与讨论

### 2.1 色谱固定相的表征

#### 2.1.1 红外光谱

首先对SiO_2_和SiO_2_@P(St-b-AA)进行了傅里叶红外光谱测试,测试结果如图2a所示。由于聚(苯乙烯-丙烯酸)共聚物的引入,与SiO_2_相比,SiO_2_@P(St-b-AA)固定相在1715 cm^-1^处出现了归属于丙烯酸的C=O特征吸收峰,在1600 cm^-1^处出现了归属于苯乙烯的C=C特征吸收峰;此外,1452 cm^-1^、703 cm^-1^处分别是烷烃和芳香烃的C-H面内弯曲振动峰,2853 cm^-1^处是C-H伸缩振动吸收峰。这些特征峰的出现表明在硅球上成功键合了聚(苯乙烯-丙烯酸)共聚物。

**图2:**
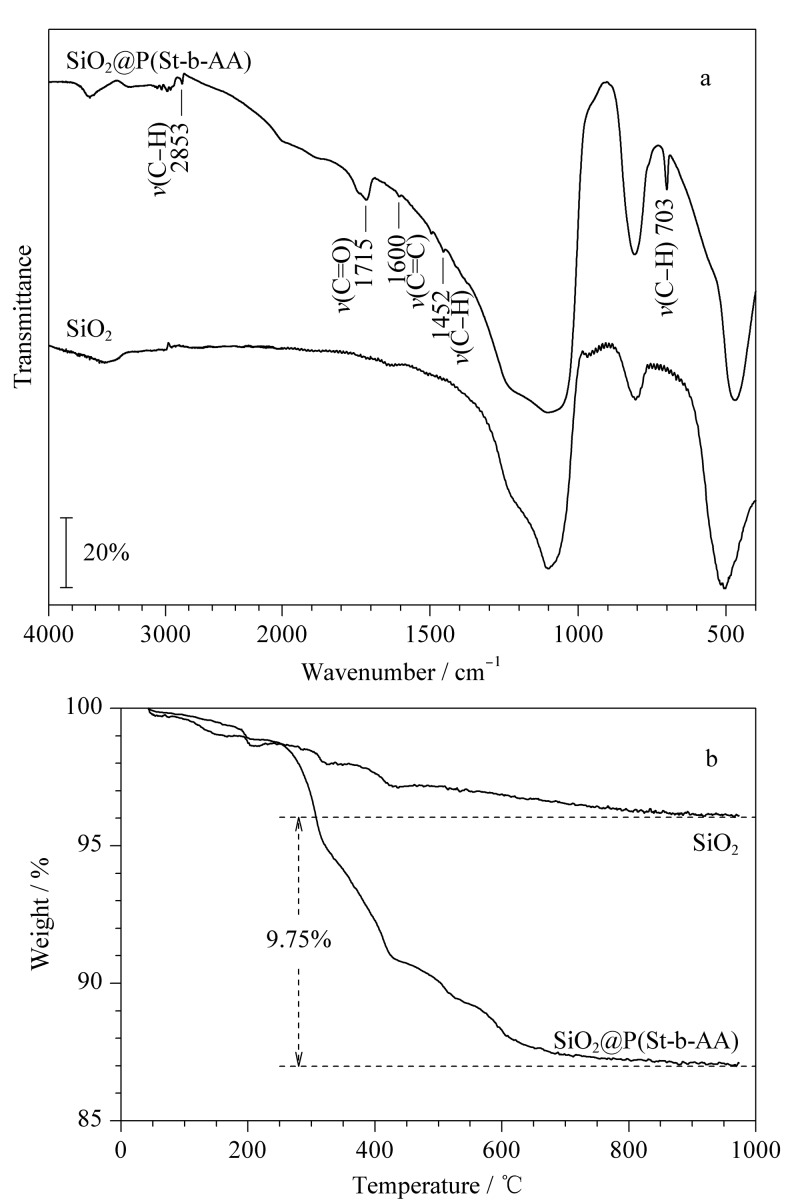
SiO_2_和SiO_2_@P(St-b-AA)的(a)红外光谱和(b)热重分析曲线

#### 2.1.2 热重分析

采用热重分析仪对固定相上聚合物的键合量进行分析。从图2b中可以看出,SiO_2_和SiO_2_@P(St-b-AA)在200 ℃内仅有少量来自于物理吸附水的失重;随着温度升高,与SiO_2_相比,SiO_2_@P(St-b-AA)在200~1000 ℃内大约有9.75%的质量损失,该损失归属于聚合物的热分解,热重结果表明了聚(苯乙烯-丙烯酸)共聚物在硅胶上成功键合并且具有良好的热稳定性。

#### 2.1.3 形貌表征

为了观察聚合物改性前后硅球的表面形态变化,采用场发射扫描电镜对SiO_2_和SiO_2_@P(St-b-AA)的形貌进行了表征,结果如图3所示。相比于未改性的SiO_2_(见图3a),改性后的硅球大小和形态均未发生改变,硅球周围和表面也无明显的聚合物和硅球碎片出现,固定相仍然保持良好的球形结构和单分散形态(见图3b)。为了考察高压装填下固定相的机械稳定性,在50 MPa压力下对SiO_2_@P(St-b-AA)固定相进行了多次装填,图3c和图3d分别为装填1次和反复装填3次后色谱填料的扫描电镜图。可以看出,装填1次后的硅球形态几乎没有发生变化;反复装填3次后,虽然硅球周围有少量碎片出现,但大部分硅球仍保持完整的球形结构,表明所制备的色谱固定相具有良好的抗压强度,在高压下不易变形,满足色谱分离测试条件。

**图3:**
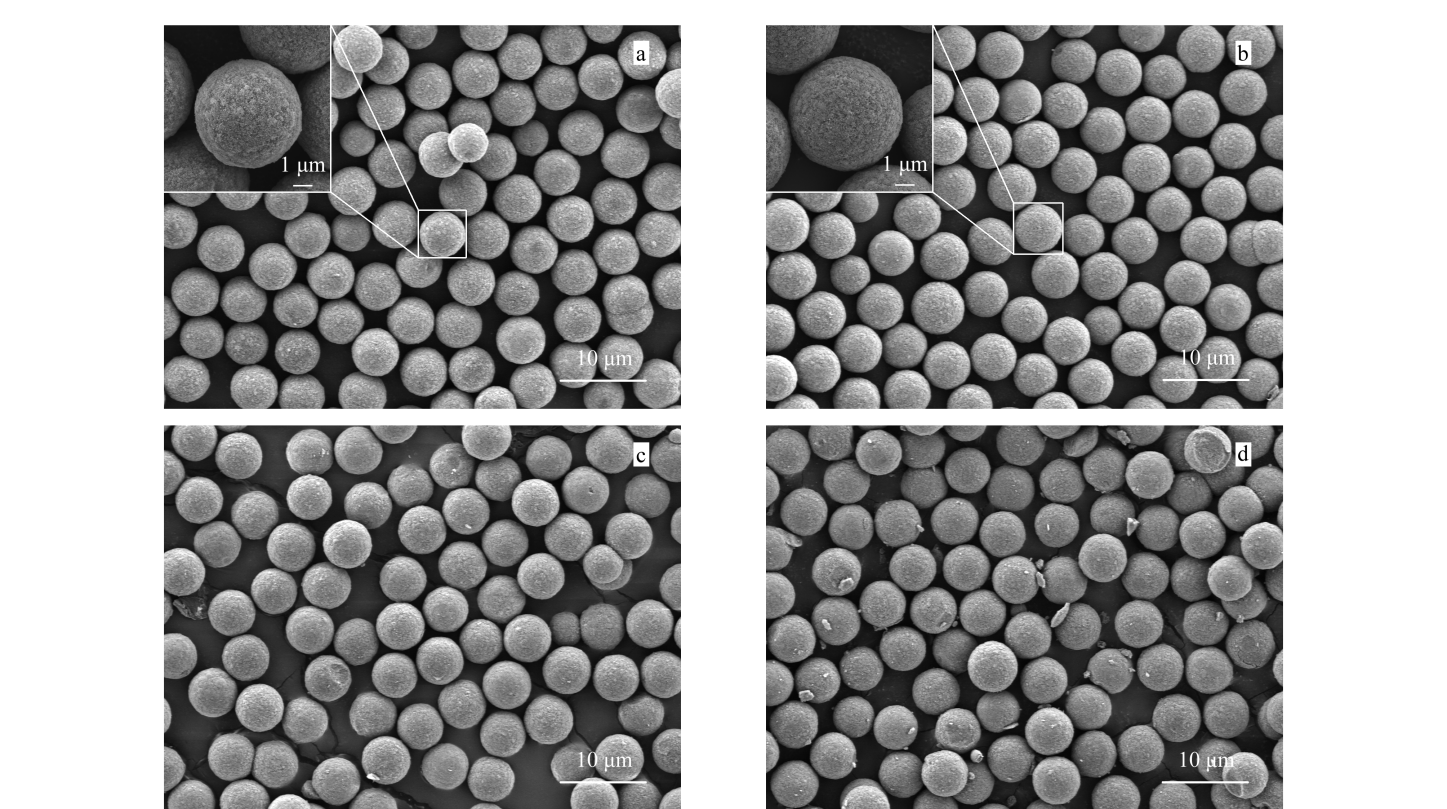
(a)SiO_2_, (b)SiO_2_@P(St-b-AA), 50 MPa压力下(c)装填1次及(d)装填3次后SiO_2_@P(St-b-AA)的扫描电镜图

#### 2.1.4 孔结构分析

SiO_2_和SiO_2_@P(St-b-AA)的N_2_吸附-脱附等温线和孔径分布图分别如图4a、4b所示。其中SiO_2_与SiO_2_@P(St-b-AA)的N_2_吸附-脱附等温线均属于Ⅳ型等温线,表明聚合物键合没有改变硅球的介孔结构,改性后的固定相孔径和孔容分别从9.76 nm、0.833 cm^3^/g下降到9.74 nm、0.743 cm^3^/g。同时,从采用BJH方法(Barret-Joyner-Halenda法)计算出的孔径分布图中可以看出,改性后固定相的孔分布均匀,表明聚合物均匀地键合到了SiO_2_表面和孔道内部,这与前面的形貌表征结果相符。

**图4:**
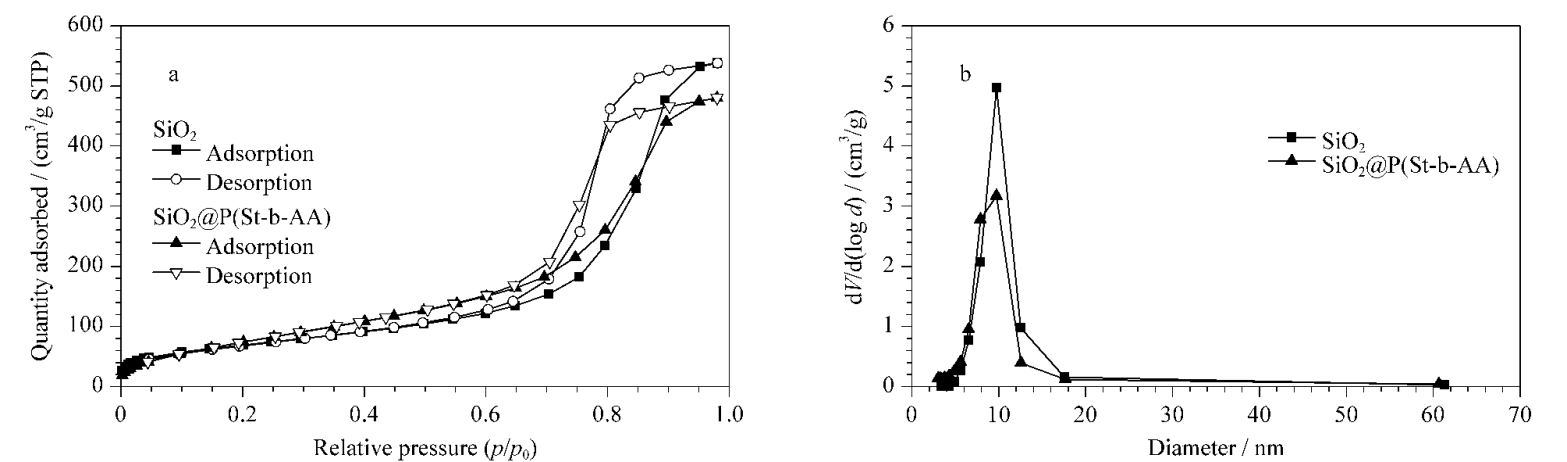
SiO_2_和SiO_2_@P(St-b-AA)的(a)N_2_吸附-脱附等温线及(b)BJH孔径分布图

#### 2.1.5 Zeta电势分析

由于固定相表面丙烯酸重复单元的存在,在pH>3.8时,羧酸基团解离使固定相带负电荷。为了考察固定相表面的带电情况,分别在25 ℃, pH=3.0、5.0和7.5的水溶液条件下对固定相表面的Zeta电势进行了测定。结果显示3个pH条件下,SiO_2_@P(St-b-AA)固定相表面的Zeta电势分别为-0.728、-7.90、-17.7 mV。随着pH由酸性变化到中性,SiO_2_@P(St-b-AA)固定相表面的电势逐渐降低,显示出带负电荷的特性。固定相表面Zeta电势的变化结果表明,在分离过程中可以通过调节流动相的pH来调控离子型化合物的分离。

### 2.2 固定相性能评价

#### 2.2.1 反相分离模式

由于SiO_2_@P(St-b-AA)固定相上具有可以提供疏水和*π-π*相互作用的苯环基团。因此,对所制备的固定相在反相模式下的分离性能进行探究具有重要意义。首先,我们选用5种烷基苯(甲苯、乙苯、丙苯、正丁基苯、戊苯)作为疏水性溶质,对SiO_2_@P(St-b-AA)固定相的保留行为和分离性能进行了评价。由图5a所示,在反相模式下,当甲醇的体积分数从40%增加到80%时,5种烷基苯的保留因子逐渐降低,符合反相模式下的分离特征。5种烷基苯在本工作所制备的PSA色谱柱及实验室内自制的Amide和C18色谱柱上的分离谱图如图5b所示。其中,在甲醇-水(50∶50, v/v)流动相条件下,在本工作制备的PSA柱上,5种烷基苯在20 min内可以实现良好的分离,且洗脱顺序与分析物的疏水性相符;在相同的洗脱条件下,Amide柱不能对5种烷基苯实现分离;而在甲醇-水(75∶25, v/v)流动相条件下,C18柱虽然能分开5种烷基苯,但保留时间较长,根据C18柱的保留机理,其在更高的水含量流动相条件(如甲醇-水,50∶50, v/v)下,保留时间将更长。

**图5:**
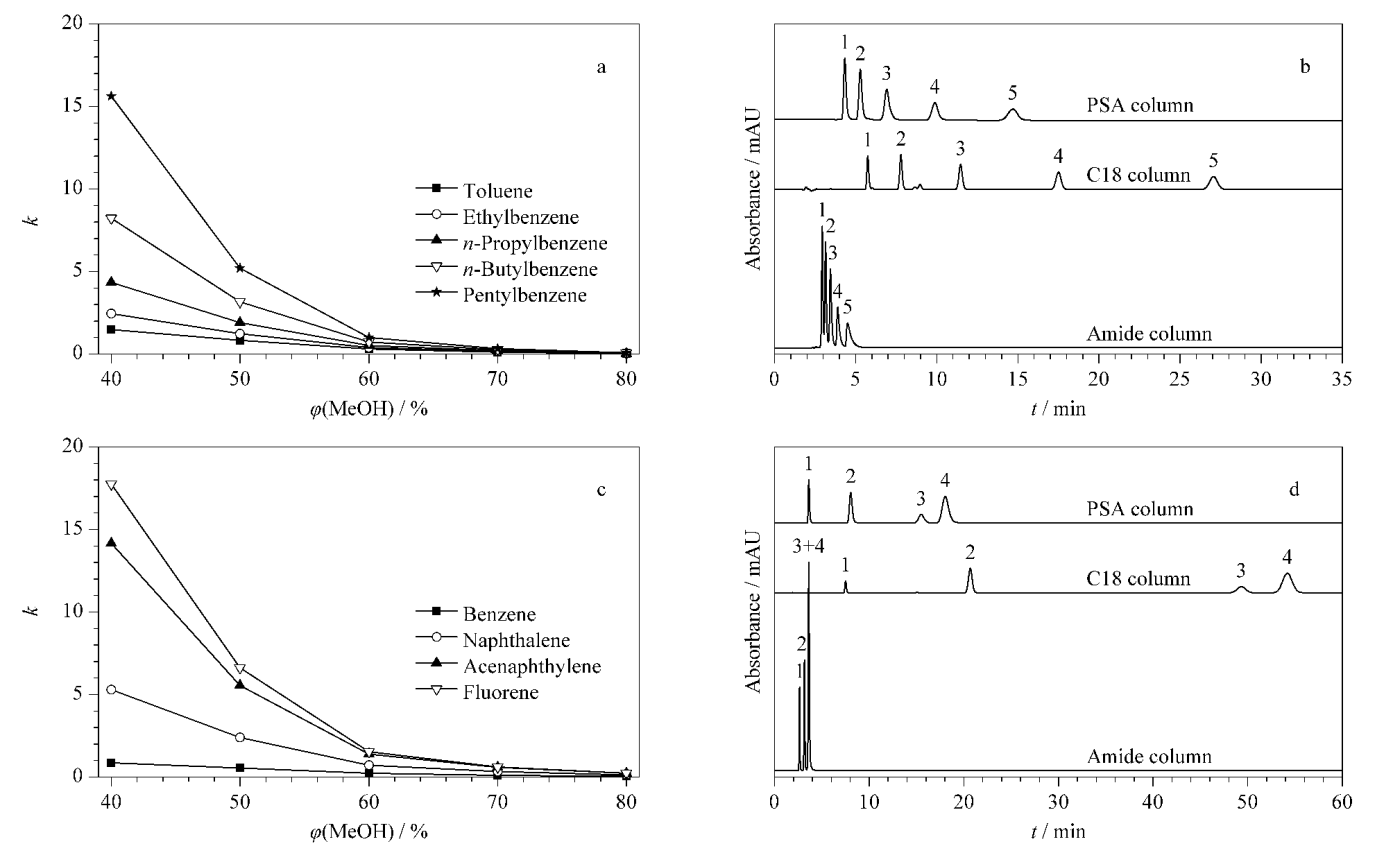
疏水性分析物在反相模式下的保留及分离评价

为了进一步探究该固定相的反相分离特征,本工作还选取了4种多环芳香烃(苯、萘、苊、芴)作为探针,测定了它们在SiO_2_@P(St-b-AA)固定相上的保留行为。图5c、5d分别显示了4种多环芳香烃保留因子随甲醇含量的变化规律及其混合物在PSA柱、Amide柱和C18柱上的分离谱图。与预期的结果一致,多环芳香烃在PSA柱上的分离规律与烷基苯相同。

整体来说,与Amide柱相比,PSA柱上由于苯环的存在表现出明显的疏水和*π-π*相互作用,可以对烷基苯和多环芳香烃进行良好的分离;与C18柱相比,PSA柱可以在体积分数更低(50%)的甲醇体系下对疏水物质进行短时间分离,有利于节约流动相成本。反相模式下的分离结果表明,本工作制备的SiO_2_@P(St-b-AA)固定相在分离疏水混合物上具有巨大潜力,可应用于反相模式下苯类同系物的分离。

#### 2.2.2 亲水分离模式

由于SiO_2_@P(St-b-AA)固定相上的羧酸基团可以为固定相与极性分析物之间提供亲水相互作用,为了探究亲水模式下该固定相的分离性能,在乙腈和甲酸铵缓冲液体系下,本实验探究了7种亲水分析物——核苷(尿苷、胞苷)、碱基(尿嘧啶、5-氟尿嘧啶、胞嘧啶、5-氟胞嘧啶)和乳清酸——在SiO_2_@P(St-b-AA)固定相上的保留特征。图6a显示了7种亲水分析物的保留因子随流动相中乙腈含量的变化情况。实验结果表明,7种分析物的保留因子均随着乙腈含量的增加而增加,在SiO_2_@P(St-b-AA)固定相上的保留符合亲水相互作用保留机制^[31]^。亲水分析物在Amide柱、C18柱和PSA柱上的分离谱图如图6b所示。其中,C18柱由于没有极性基团无法对亲水物质进行分离;Amide柱对尿苷和5-氟胞嘧啶的分离度不够;而PSA柱可以在15 min内对7种分析物实现基线分离,并且峰形良好。此外,7种亲水物质在PSA柱上的洗脱顺序与其极性强度并不一致,这说明亲水物质在SiO_2_@P(St-b-AA)固定相上的保留机理不只是单一的亲水相互作用,固定相与溶质之间还存在氢键等相互作用力。

**图6:**
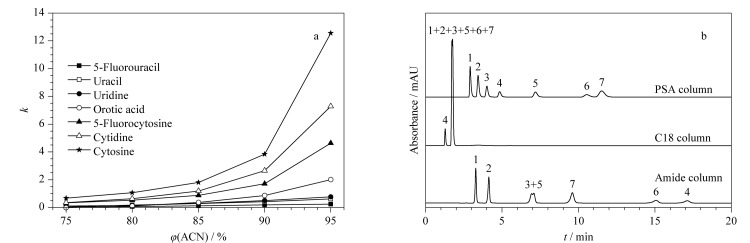
亲水性分析物在亲水模式下的保留及分离评价

#### 2.2.3 离子交换分离模式

SiO_2_@P(St-b-AA)固定相上的羧酸基团除了提供亲水相互作用之外,流动相的pH还会影响羧酸基团解离,因此,SiO_2_@P(St-b-AA)固定相还将为离子型化合物提供静电相互作用。为了探究固定相的离子交换能力,本工作考察了流动相中水相pH值在3.2~7.5范围内变化时,固定相对4种有机碱和4种有机酸保留的影响,其中pH<5.3时,溶液pH使用甲酸调节,随后加入三乙胺调节至中性。4种有机碱和4种有机酸的结构式及p*K*_a_见图7。

**图7:**
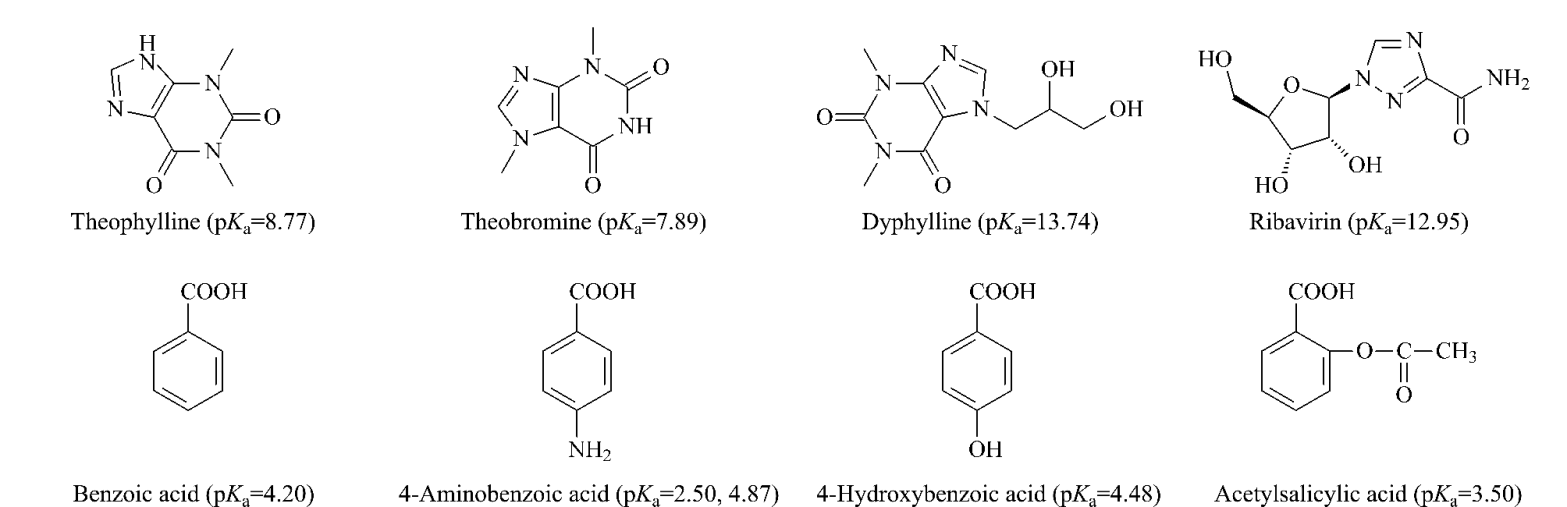
4种有机碱和4种有机酸的结构式及p*K*_a_

图8a显示了4种有机碱保留时间随pH的变化情况。其中除了利巴韦林(ribavirin)的保留时间在pH由4.2增加到7.5时明显增加外,茶碱(theophylline)、可可碱(theobromine)和二羟丙茶碱(dyphylline)的保留时间无明显变化。从4种有机碱的结构式中可以发现后3种有机碱的结构相似,均具有类似的并苯结构,而利巴韦林具有多个羟基结构。多羟基结构更容易与固定相之间产生亲水相互作用,也更容易受到流动相中水相pH的影响,因此随着pH增大,带正电荷的利巴韦林与带负电荷的固定相之间逐渐增强的相互吸引力导致保留增强;而茶碱、可可碱和二羟丙茶碱在固定相上的保留还受到苯环*π-π*相互作用的影响,水相pH变化对其保留的影响被削弱。流动相中乙腈与水的体积比对4种有机碱的分离结果也表明水相的体积比更易对利巴韦林的保留产生影响(图8b)。

**图8:**
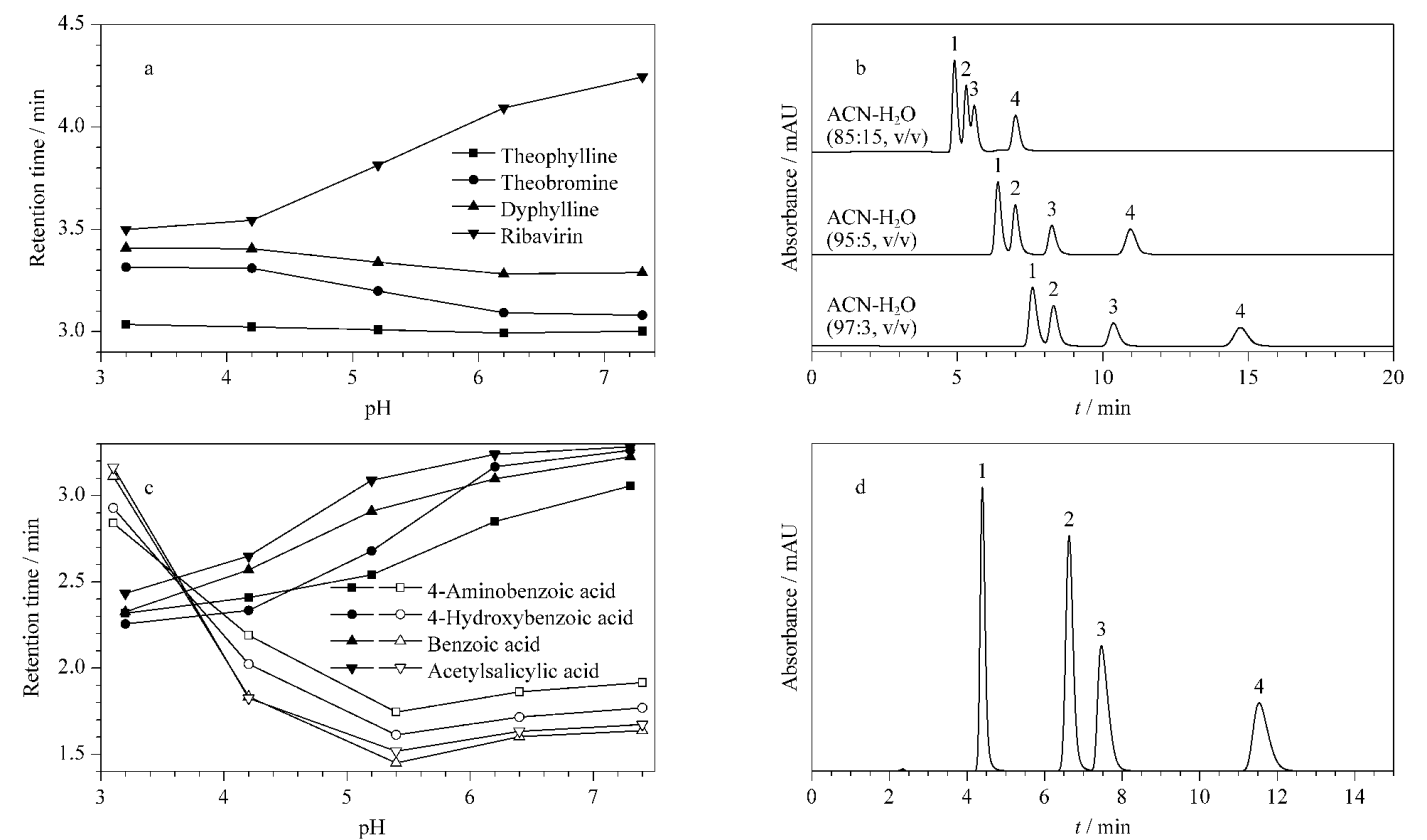
4种有机碱、4种有机酸保留时间随流动相pH的变化及其在PSA色谱柱上的分离色谱图

为了进一步探究固定相对离子型化合物的保留机理,选取有机酸探究了固定相对酸性物质的保留作用。如图8c所示,以100 mmol/L甲酸铵缓冲液作为水相流动相,水相pH由3.2逐渐增加到7.5时,4种有机酸的保留时间逐渐增加;而以纯水作为水相流动相时,随着水相pH的增加,有机酸的保留时间呈现先下降后上升的变化规律。当以甲酸铵缓冲液作为流动相时,酸性化合物电离出来的负离子容易先与缓冲液中的N $H_{4}^{+}$ 发生结合产生络合物,并且与带负电荷的固定相产生静电吸附作用,随着pH增大,静电吸附作用力增强导致分析物的保留时间增大。以纯水作为流动相则屏蔽了N $H_{4}^{+}$ 的作用。随着pH增加,分析物与固定相之间的静电排斥作用逐渐增强,保留时间缩短;但当pH>5.3时,保留时间略有增加,这是因为pH调节剂三乙胺的加入削弱了这种相互作用。在乙腈-100 mmol/L NH_4_FA(97∶3, v/v)流动相条件下,4种有机酸的分离谱图如图8d所示。

综上所述,有机碱和有机酸在SiO_2_@P(St-b-AA)上的保留行为表明所制备的固定相带有明显的负电荷,能与溶质分子发生静电相互作用,在分离碱性样品时表现出一定的阳离子交换能力,分离酸性样品时表现出静电排斥作用。但该固定相对酸碱化合物的保留还受到其他作用的影响,表明固定相与溶质之间的静电相互作用较弱。

### 2.3 混合样品分离的稳定性及制备方法的重复性

使用具有混合分离模式的色谱固定相同时分离多种不同性质的分析物至关重要,本实验选取甲苯、尿苷、胞苷、苯甲酸、利巴韦林5种物质作为混合样品,在PSA色谱柱上对其进行了分离,并对其分离的日内和日间稳定性进行了考察,结果如图9a所示。在同一色谱条件下,日内连续进样6次,保留时间的RSD值为0.10%~1.0%;连续4天重复进样,保留时间的RSD最大值为0.85%(见表1)。良好的日内进样稳定性和日间重复性表明SiO_2_@P(St-b-AA)固定相在不同极性组分分析物的分离中具有良好的应用潜力。

**图9:**
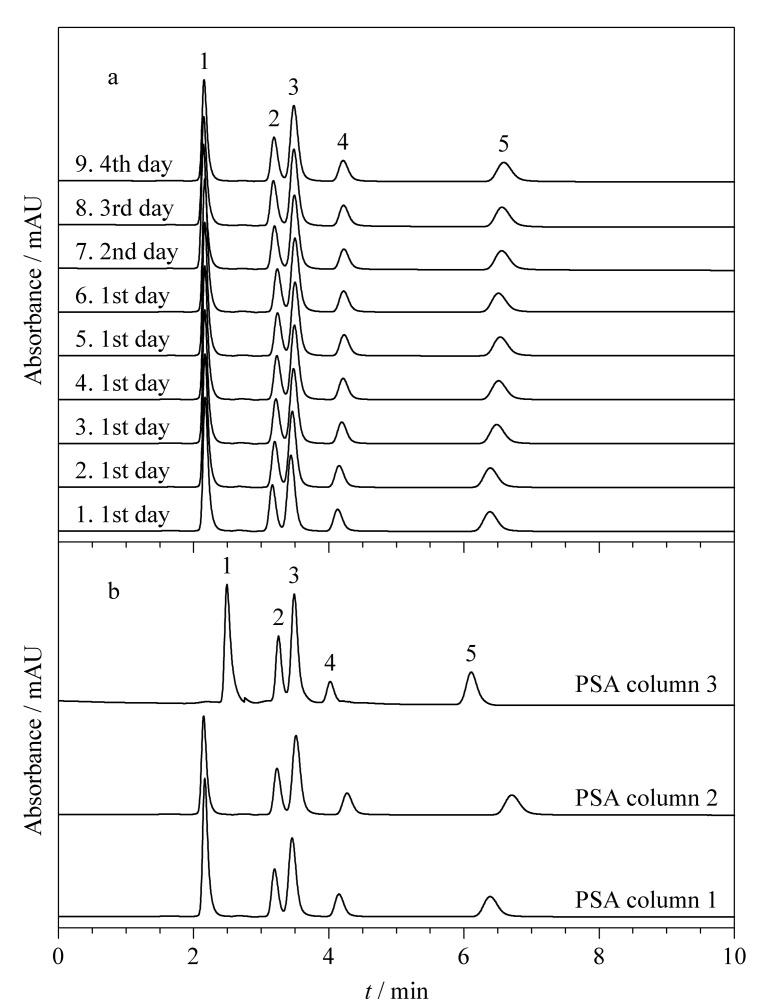
(a)PSA色谱柱的进样稳定性及(b)SiO_2_@P(St-b-AA)合成过程的批次重复性

**表1:** 混合样品在PSA色谱柱上的连续进样稳定性和日间稳定性

No.	Analyte	RSDs/%	
		Intra-day stability (*n*=6)	Inter-day stability (*n*=4)
1	toluene	0.10	0.39
2	benzoic acid	0.92	0.85
3	uridine	0.75	0.48
4	ribavirin	0.95	0.18
5	cytidine	1.0	0.33

为了考察“一锅法”制备方法的重复性,在相同的实验条件下合成了另外两锅SiO_2_@P(St-b-AA)固定相。热重结果显示3个批次制备的固定相在200~1000 ℃内质量损失相近,分别为12.28%、13.23%和11.66%;此外,通过分离5种混合样品对这3个批次固定相装填的色谱柱进行了测试,结果如图9b所示,3根色谱柱在对不同极性组分样品的分离上表现出相近的分离度和峰形,每根柱上分析物保留时间的微小差异可能是柱填充过程中的偏差造成的。总体而言,简便的“一锅法”制备的SiO_2_@P(St-b-AA)固定相具有良好的制备批次稳定性。

## 3 结论

本工作采用“一锅法”成功地在硅胶表面键合上了聚(苯乙烯-丙烯酸)共聚物,制备了具有混合分离性能的SiO_2_@P(St-b-AA)固定相。分别采用疏水、亲水性分析物对固定相的保留机理和分离性能进行了评价,结果表明所制备的固定相具有良好的反相和亲水保留特性,分离性能优于自制的C18固定相和Amide固定相;对碱性溶质的分离还证明了该固定相具有一定的弱阳离子交换能力。在混合样品的分离上该固定相表现出良好的分离性能和重复性;制备方法的重复性考察表明SiO_2_@P(St-b-AA)固定相的合成还具有良好的制备批次稳定性。这些结果表明本文所采用的“一锅法”制备方法是可行的,为其他多种聚合物改性硅基固定相的制备提供了一种新思路,具有良好的应用前景。此外,由于本工作所制备的固定相对离子型化合物的保留机理较为复杂,更多种类离子型化合物在该固定相上的保留行为以及硅胶上苯环和羧酸基团键合量的优化工作值得进一步探究。
